# Mast Cell-Derived Histamine Mediates Cystitis Pain

**DOI:** 10.1371/journal.pone.0002096

**Published:** 2008-05-07

**Authors:** Charles N. Rudick, Paul J. Bryce, Laura A. Guichelaar, Ruth E. Berry, David J. Klumpp

**Affiliations:** 1 Department of Urology, Feinberg School of Medicine, Northwestern University, Chicago, Illinois, United States of America; 2 Department of Microbiology-Immunology, Feinberg School of Medicine, Northwestern University, Chicago, Illinois, United States of America; 3 Division of Allergy-Immunology, Feinberg School of Medicine, Northwestern University, Chicago, Illinois, United States of America; Centre de Recherche Public-Santé, Luxembourg

## Abstract

**Background:**

Mast cells trigger inflammation that is associated with local pain, but the mechanisms mediating pain are unclear. Interstitial cystitis (IC) is a bladder disease that causes debilitating pelvic pain of unknown origin and without consistent inflammation, but IC symptoms correlate with elevated bladder lamina propria mast cell counts. We hypothesized that mast cells mediate pelvic pain directly and examined pain behavior using a murine model that recapitulates key aspects of IC.

**Methods and Findings:**

Infection of mice with pseudorabies virus (PRV) induces a neurogenic cystitis associated with lamina propria mast cell accumulation dependent upon tumor necrosis factor alpha (TNF), TNF-mediated bladder barrier dysfunction, and pelvic pain behavior, but the molecular basis for pelvic pain is unknown. In this study, both PRV-induced pelvic pain and bladder pathophysiology were abrogated in mast cell-deficient mice but were restored by reconstitution with wild type bone marrow. Pelvic pain developed normally in TNF- and TNF receptor-deficient mice, while bladder pathophysiology was abrogated. Conversely, genetic or pharmacologic disruption of histamine receptor H1R or H2R attenuated pelvic pain without altering pathophysiology.

**Conclusions:**

These data demonstrate that mast cells promote cystitis pain and bladder pathophysiology through the separable actions of histamine and TNF, respectively. Therefore, pain is independent of pathology and inflammation, and histamine receptors represent direct therapeutic targets for pain in IC and other chronic pain conditions.

## Introduction

IC is a chronic bladder inflammatory disease with unknown etiology that afflicts as many as 1 million patients in the United States. IC is associated with severe pelvic pain and voiding dysfunction that includes urinary frequency, urgency, and nocturia [1], [2], [3]. Clinical studies demonstrate elevated mast cell numbers in the lamina propria of IC bladder biopsies, and the partial efficacy of neuromodulatory therapies suggests neural-immune interactions play a role in IC pathogenesis ([4], [5] and reviewed in [1]). Mast cells contain preformed stores of immune mediators, such as histamine and TNF, and can be activated to release these stores by neurotransmitters such as substance P. These observations have suggested a central role for mast cells in IC pathogenesis whereby activation of bladder-associated circuits in the central nervous system (CNS) initiates substance P release by peripheral nerves in the bladder that then promotes substance P-mediated mast cell activation [6]. This mast cell activation, in turn, is postulated to induce bladder inflammation by acting on urothelium, the epithelium that lines the bladder. In support of this hypothesis, murine cystitis models have demonstrated a requirement for mast cells and substance P receptors in bladder inflammation [7], [8]. Also, increased levels of urinary histamine metabolites have been detected in IC patient urines, and accumulation of lamina propria mast cells is correlated with IC symptoms [4], [5], [9], [10]. However, other inflammatory markers are inconsistent features of IC, and the basis of pelvic pain is unclear. Here we examine mechanisms of pelvic pain in an established murine model that recapitulates key aspects of IC, including lamina propria mast cell accumulation and pelvic pain [11], 12, 13.

The attenuated Bartha's strain of pseudorabies virus (PRV) is an α-herpesvirus that is taken up by neurons and undergoes retrograde transport and viral replication within the CNS. PRV was originally shown to cause cystitis in rats when injected into the tailbase *abductor caudalis dorsalis* muscle and taken up by motor neurons. PRV-induced cystitis is a neurally mediated event triggered by viral action in the CNS, and the cystitis is associated with bladder mast cell activation, even though Bartha's PRV is incapable of descending sensory nerves to the bladder [11], [13], [14], [15], [16]. In mice, PRV causes urothelial expression of RANTES (*R*e-*A*ctivated in *N*ormal, *T* cell-*E*xpressed, *S*ecreted), a chemokine known to promote mast cell trafficking [11], [12]. RANTES drives mast cell accumulation in the lamina propria that juxtaposes activated mast cells with the urothelium, and the proximity of mast cells to the urothelium induces mast cell-dependent, TNF-dependent bladder pathophysiology. This bladder pathophysiology includes the formation of apoptotic lesions in the urothelium and a marked loss of trans-epithelial resistance (TER) that is normally characteristic of urothelial barrier function in the intact bladder [12], [17]. Thus, the pathophysiology of murine PRV cystitis is consistent with human IC where the presence of urothelial lesions in patient biopsies correlates with IC symptoms, and many IC patients are exquisitely sensitive to instillation of nerve-depolarizing concentrations of KCl into the bladder, a finding that suggests a loss of barrier function [18].

Murine PRV cystitis was also recently shown to induce bladder-associated pain specific to the pelvic region in female C57BL6/J mice [13]. Although inflammation was restricted to the bladder, the bladder-associated pelvic pain was blocked by instillation of 2% lidocaine into either the bladder or colon, indicating modulation of pelvic pain responses through organ crosstalk by visceral inputs distinct from the site of pathophysiology. Since we previously showed that mast cells mediate the bladder pathophysiology of murine PRV cystitis, we hypothesized that pelvic pain is also mediated by mast cells. In this study, both pelvic pain and bladder pathophysiology were dependent upon mast cells. TNF mediated cystitis pathophysiology, while pelvic pain developed independent of TNF. Conversely, genetic or pharmacologic disruption of histamine receptors H1R or H2R attenuated pelvic pain without altering pathophysiology. These data demonstrate that cystitis pain and bladder pathophysiology are dependent on mast cells through the independent actions of histamine and TNF, respectively, and identify histamine receptors as therapeutic targets for direct intervention in pelvic pain of IC.

## Results

### PRV induces pelvic pain

**Figure 1:**
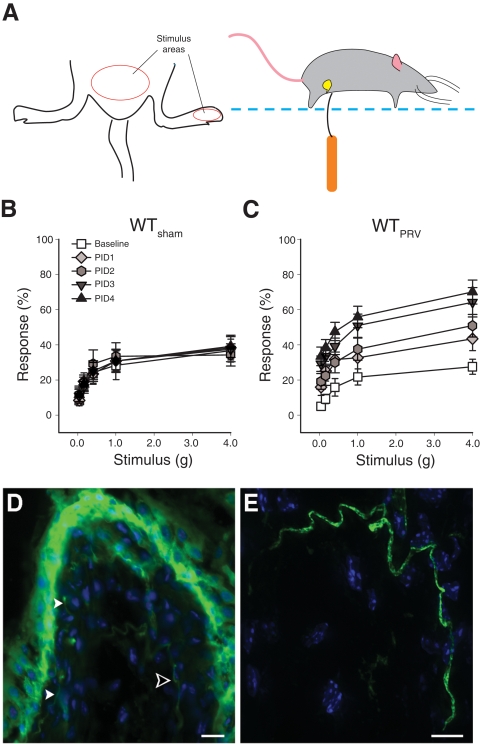
PRV induces pelvic pain. A) Tactile sensitivity was assessed by stimulating the suprapubic and hindpaw skin with von Frey filaments (left panel) of a mouse placed into a chamber with a mesh floor (right panel). Referred visceral hyperalgesia was measured in female mice as responses to mechanical stimulation of the pelvic region using von Frey filaments of 5 calibrated forces. Data represent the mean response to ten successive applications for each fiber ±SEM at baseline and at PID 1, 2, 3, and 4 following infection with 2.3×10^6^ pfu Bartha's PRV in the tailbase muscle. B) Responses to pelvic stimulation of C57BL/6J mice sham-infected with inactivated PRV (n = 12). The symbol key shown in (B) also applies to panel (C). C) Responses to pelvic stimulation of C57BL/6J mice infected with PRV reveal enhanced sensitivity to suprapubic stimuli (n = 12). ANOVA indicated a significant increase in responses from baseline at all filaments tested in PRV-treated mice at PID 2, 3 and 4 (P<0.05). D) Fluorescein-isolectin B4 staining (green) of bladder tissue reveals processes in the lamina (arrowheads). E) A single isolectin-stained fiber (open arrowhead in D) is shown at higher magnification. The mean diameter is 1.31 µm along its length of 1071 µm. Scale bars represent 16 µm and 10 µm for D and E, respectively.

Pain originating from a visceral organ is typically referred to a corresponding “dermatome” on the skin that shares spinal innervation with the given visceral organ [19]. Therefore, to quantify bladder-associated pelvic pain, we assessed tactile sensitivity of the pelvic region in response to stimulation by von Frey filaments [13], [20]. Mechanical stimulation of the pelvic area of female C57BL/6J (B6) mice evoked baseline responses (pelvic withdrawal, jumping, or pelvic licking/scratching) where the percentage of responses increased as a function of force applied using graded filaments (Fig. 1A). Following PRV infection, mice exhibited a progressively enhanced sensitivity to pelvic stimulation consistent with the development of pelvic pain that became significant by post-infection day 2 (PID 2; Fig. 1C, P<0.05), but pain responses did not develop in sham-infected mice (Fig. 1B). These data indicate PRV-induced pelvic tactile hypersensitivity, or “allodynia.” To assess the specificity of PRV-induced tactile allodynia, we also quantified the 50% threshold sensitivity in the paw [21], [22]. PRV induced no changes in tactile sensitivity of the plantar region of the hind paw of any groups tested (Table S1). These data indicate that PRV induces a progressive pain specific to the pelvic region, consistent with previous observations [13].

To understand the basis of bladder-associated pain, we next examined nerve fibers in the mouse bladder. Since C fibers are the nociceptors associated with chronic pain, bladder sections were stained with isolectin B4, a marker for C fibers that stains most bladder nociceptor afferents [23], [24], [25]. In addition to labeling of the urothelium, a tissue layer heavily decorated with carbohydrate structures, isolectin B4 also labeled fibers that were evident in the lamina propria (Figs. 1D and E and Movie S1). Image analysis of isolectin B4-positive fibers within the lamina propria revealed a mean diameter of 1.089±0.095 µm with a range of 0.728–1.361 µm (n = 7), consistent with the morphology of bladder C fiber nociceptor axons in dorsal root ganglia [24]. Since we previously found that PRV infection induces mast cell accumulation in the lamina propria [11], [12], [17], the location of C fibers within the bladder lamina propria raises the possibility that mast cell-C fiber interactions contribute to PRV-induced pelvic pain.

### Pelvic pain requires mast cells

**Figure 2:**
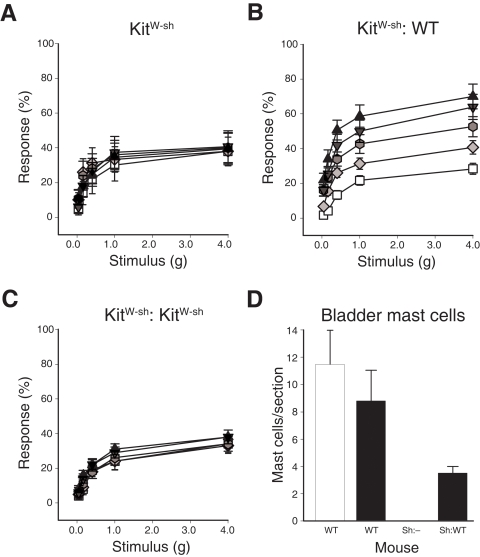
Mast cells mediate pelvic pain. A) Pelvic stimulation of mast cell-deficient *Kit^W-sh^/Kit^W-sh^* mice infected with PRV reveals no progressive tactile sensitivity (n = 15). B) *Kit^W-sh^/Kit^W-sh^* mice reconstituted with wild type B6 bone marrow exhibited progressive pelvic sensitivity (n = 15). ANOVA indicated a significant increase in response from baseline at all filaments tested in PRV-treated mice at PID 2, 3 and 4 (P<0.05). C) *Kit^W-sh^/Kit^W-sh^* reconstituted with *Kit^W-sh^/Kit^W-sh^* bone marrow did not develop progressive pelvic sensitivity (n = 10). D) Bone marrow reconstitution of *Kit^W-sh^/Kit^W-sh^* mice with B6 bone marrow restored bladder mast cells, whereas reconstitution with *Kit^W-sh^/Kit^W-sh^* bone marrow did not (data represent the mean of bladder sections from 4 (sham), 5 (PRV), 5 (*Kit^W-sh^/Kit^W-sh^*), or 4 (*Kit^W-sh^/Kit^W-sh^* mice reconstituted B6 bone marrow) mice). In all panels, error bars depict SEM. Data indicate values for sham-infected (white bar) and PRV-infected mice (black bars).

Since clinical studies suggest mast cell involvement in the pathophysiology of IC, we examined PRV-induced tactile allodynia in mast cell-deficient mice to directly investigate the role of mast cells in pelvic pain. Mast-cell deficient *Kit^W-sh^/Kit^W-sh^* mice have a non-coding mutation in the gene encoding stem cell factor, c-kit, and have proven valuable for examining the role of mast cells in disease [26], [27]. In contrast to the development of pelvic pain in wild type B6 mice, PRV infection of mast cell-deficient *Kit^W-sh^/Kit^W-sh^* mice failed to induce enhanced pelvic responsiveness above baseline, suggesting a role for mast cells in the development of pelvic pain (Fig. 2A). Induction of pelvic pain by PRV was fully restored in *Kit^W-sh^/Kit^W-sh^* mice where bladder mast cells were reconstituted by transfusion with wild type B6 bone marrow (P<0.05) but not in *Kit^W-sh^/Kit^W-sh^* mice reconstituted with *Kit^W-sh^/Kit^W-sh^* bone marrow (Figs. 2B and C, respectively). Bladder mast cells were then quantified in toluidine blue-stained bladder tissue sections, and the restoration of pelvic pain responses was indeed associated with reconstitution of bladder mast cells (Fig. 2D).

Mast cells are not likely required for general pain responses because *Kit^W-sh^/Kit^W-sh^* mice exhibited normal sensitivity to capsaicin administered to the colon (colonic capsaicin induced a 226.0±66.2% increase in pelvic responses relative to baseline in wild type B6 mice, and capsaicin induced a 235.7±88.6% increase in responses in *Kit^W-sh^/Kit^W-sh^* mice; P>0.05). To exclude the possibility that mast cell-deficiency altered pelvic pain responses by acting on general physiology or behavior, we also quantified normal behaviors during free roaming (Table S2). No significant increase in response to PRV was detected in grooming, cage crossing, or rearing in any treatment group or genetic background throughout the observation period, suggesting that the role of mast cells in pelvic pain is not due to generalized alterations in behavior that might be manifested as enhanced responsiveness to pelvic tactile stimuli. Similarly, an absence of weight changes or altered development of end-stage CNS disease in any experimental group indicates that mast cell status is not associated with dramatic changes in gross physiology or susceptibility to PRV infection (Table S3 and data not shown). Therefore, these data suggest a specific requirement for mast cells in the development of bladder-associated pelvic pain.

### TNF does not modulate pelvic pain

**Figure 3:**
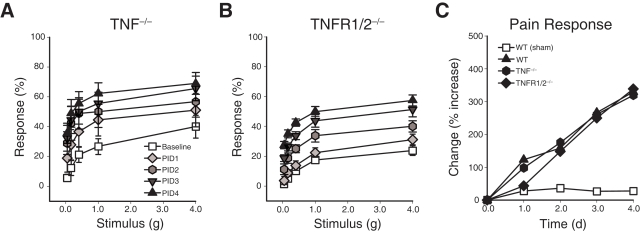
TNF does not mediate cystitis pain. A) TNF^−/−^ mice exhibit normal, progressive pelvic sensitivity following PRV infection (n = 9). B) TNFR1/2^−/−^ mice exhibit normal, progressive pelvic sensitivity following PRV infection (n = 8). In (A) and (B), error bars depict SEM. C) TNF^−/−^ and TNFR1/2^−/−^ mice exhibit overall responses to pelvic stimulation that are indistinguishable from wild type B6 mice (B6). Percent responses at each PID were calculated as total responses to all fibers relative to baseline responses. The symbol key shown in (A) applies to panels A–B.

Our previous studies demonstrated that PRV-induced bladder pathophysiology was dependent upon TNF [12], [17], so we examined the role of TNF in pelvic pain responses by quantifying pelvic tactile sensitivity in TNF^−/−^ and TNFR1/2^−/−^ mice following PRV infection. Responses to mechanical stimulation of the pelvic area were significantly greater at PID 2-4 than baseline responses in both TNF^−/−^ and TNFR1/2^−/−^ mice (Figs. 3A and B, respectively; P<0.05). Furthermore, the increase from baseline response of either TNF^−/−^ or TNFR1/2^−/−^ mice infected with PRV was indistinguishable from PRV-infected wild-type mice (Fig. 3C). These data suggest that a non-TNF mast cell factor(s) mediates pelvic pain induced by PRV.

### Histamine receptors 1 and 2 modulate pelvic pain

**Figure 4:**
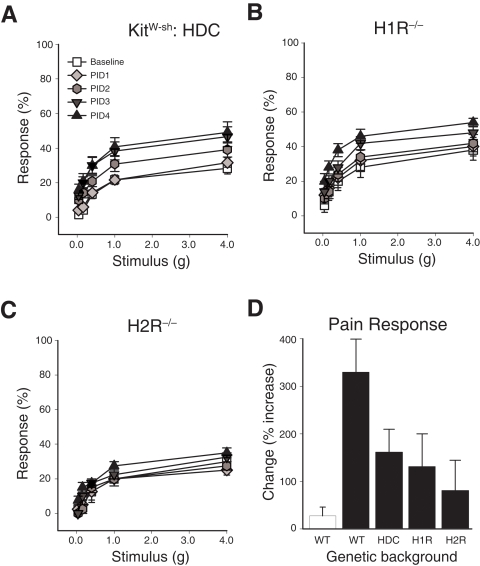
Histamine mediates cystitis pain. A) *Kit^W-sh^/Kit^W-sh^* mice reconstituted with HDC^−/−^ marrow exhibit reduced pelvic sensitivity following PRV infection (n = 12). Enhanced pelvic sensitivity relative to baseline (P<0.05) was delayed until PID3. B) H1R^−/−^ mice exhibit reduced pelvic sensitivity following PRV infection (n = 12). Enhanced pelvic sensitivity relative to baseline (P<0.05) was delayed until PID4. (n = 5). C) H2R^−/−^ mice did not develop enhanced pelvic sensitivity following PRV infection (n = 4). D) Mice deficient for HDC, H1R, or H2R exhibit diminished pelvic sensitivity at PID 4 relative to WT mice. Percent responses at PID4 calculated as total responses to all fibers relative to baseline responses for sham-infected (white bar) and PRV-infected mice (black bars). The symbol key shown in (A) applies to panels A–C. Error bars depict SEM, and significance was determined by ANOVA.

A role for histamine and histamine receptors in pain responses has been documented in both humans and animal models [28], [29], [30], [31]. However, histamine can be derived from various cell types, including mast cells, basophils, and histaminergic neurons. More recently, inflammatory cells such as neutrophils and dendritic cells have been shown to produce histamine [32], [33]. The specific cell type regulating histamine-mediated pain is unknown. To determine whether mast cell-derived histamine mediates pelvic pain, we examined pelvic sensitivity in *Kit^W-sh^/Kit^W-sh^* mice reconstituted with bone marrow of mice deficient in histadine decarboxylase (HDC), the rate-limiting enzyme in histamine biosynthesis. *Kit^W-sh^/Kit^W-sh^* mice reconstituted with HDC^−/−^ bone marrow exhibited attenuated pelvic pain responses such that HDC^−/−^-reconstituted mice did not develop significant pain until PID3 (P<0.05), whereas +/+-reconstituted mice developed significant pain by PID2 (compare Figs. 4A and 1C). We next examined pain responses in mice deficient for histamine receptor H1R or H2R to define the mechanisms by which mast cell-derived histamine induces pelvic pain (Figs. 4B & C). H1R^−/−^ mice failed to develop significant pelvic pain until PID4 (P<0.05), whereas PRV-induced pain was completely abrogated in H2R^−/−^ mice. Comparing overall pain development, HDC^−/−^-reconstituted mice developed pelvic pain that was diminished 55.7% relative to B6 wild type mice, and H1R^−/−^ and H2R^−/−^ mice developed only 34.3% and 17.6% of normal pelvic pain, respectively (Fig. 4D). Therefore, both H1R and H2R play roles in bladder-associated pelvic pain.

### Antihistamines attenuate PRV-induced bladder pain

**Figure 5:**
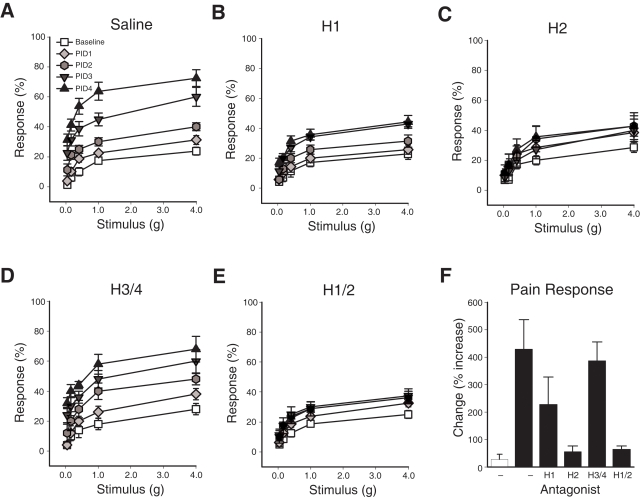
Antihistamines attenuate cystitis pain. A) Wild type B6 mice treated by oral gavage with saline exhibited significantly increased pelvic sensitivity relative to baseline at PID 2, 3 and 4 for all filaments tested (n = 8, P<0.05). B) B6 mice treated by oral gavage with the H1R antagonist diphenhydramine showed reduced pelvic sensitivity that was not significantly increased above baseline until PID 3 (n = 7, P<0.05). C) B6 mice treated by oral gavage with H2R antagonist ranitidine did not develop significant pelvic sensitivity (n = 7). D) B6 mice treated by oral gavage with the H3R/H4R antagonist thioperamine developed pelvic pain similar to saline treated mice that was significant at PID2-4 (n = 5, P>0.05). E) Treatment of B6 mice with a diphenhydramine/ranitidine dual therapy reduced pelvic pain responses to a similar extent as in mice treated with ranitidine alone (n = 8). F) The percent increase from baseline in response to pelvic stimulation at PID4 for sham-infected (white bar) and PRV-infected mice (black bars). The symbol key shown in (A) applies to panels A–E. Error bars depict SEM, and significance was determined by ANOVA.

Pilot clinical studies suggest that antihistamine therapy improves IC-related pelvic pain, and the mechanism for this effect is unknown [1]. Quantitative RT-PCR revealed that histamine receptors 1, 2, 3 and 4 are expressed in the bladder and the colon (Fig. S1). To examine histamine receptors as therapeutic targets for pelvic pain, we examined the effects of pharmacologic inhibitors on the development of PRV-induced pain responses. PRV-infected C57BL6/J mice were treated by oral gavage with saline or with antihistamines specific for H1R (diphenhydramine), H2R (ranitidine), or H3R/H4R (thioperamide) by gavage. Mice treated with saline alone exhibited responses to pelvic stimuli that were significantly greater than baseline by PID 2, 3 & 4 (Fig. 5A; P<0.05) and were similar to wild-type mice infected with PRV (compare with Fig. 1C). In contrast, diphenhydramine-treated mice failed to develop significant pain until PID 3 and 4 and developed only 49.9% of the total pain exhibited by saline-treated mice (Fig. 5B, P<0.05, and Fig. 5F), consistent with the reduced pelvic pain in H1R^−/−^ mice. Similar to the findings in H2R^−/−^ mice, ranitidine-treated wild type mice showed no significant pain responses relative to baseline at any timepoint and developed only 7.1% of the pain exhibited by saline-treated mice (Figs. 5C and 5F). Mice treated with a diphenhydramine/ranitidine cocktail showed similarly reduced pain compared to mice treated with ranitidine alone, with total pain score of 9.3% of pain exhibited by saline-treated mice (Figs. 5E and F). The effects of anti-histamine therapy were specific for H1R and H2R, for thioperamine-treated mice developed significant pelvic pain that was 90.2% of pain in mice receiving saline (Fig. 5D, P>0.05, and Fig. 5F). The efficacy of anti-H1R and anti-H2R therapies was not likely due to non-specific effects of these drugs on pelvic sensitivity or behavior because no significant change in baseline tactile sensitivity or free-roaming behaviors were detected (Fig. 3 and Tables S1 and S2). Therefore, these data suggest that mast cell-dependent pelvic pain is mediated by histamine acting via H1R and H2R.

### Histamine does not contribute to bladder pathophysiology

**Figure 6:**
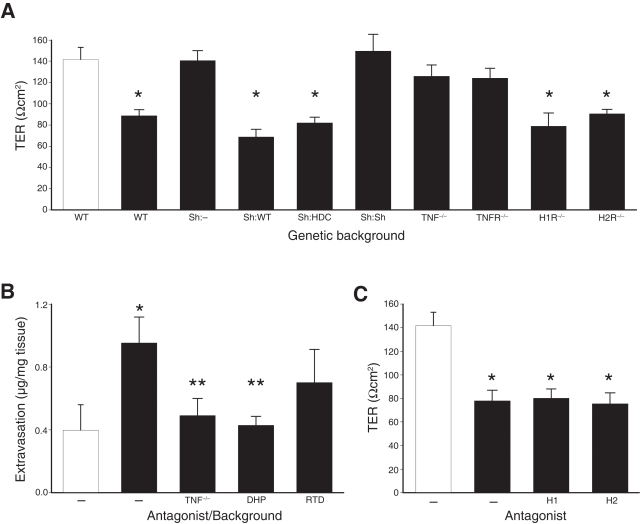
Pathophysiology and inflammation are separable from cystitis pain. A) Bladder TER was quantified ex vivo for tissues of sham-infected B6 mice (white bar) or PRV-infected mice (black bars). PRV infection significantly decreased TER in B6 mice (WT, n = 8, *P<0.05), *Kit^W-sh^/Kit^W-sh^* mice reconstituted with wild type (Sh:WT, n = 11, *P<0.05) or HDC^−/−^ bone marrow (Sh:HDC, n = 12, *P<0.05), H1R^−/−^ mice (n = 5, *P<0.05), or H2R^−/−^ mice (n = 4, *P<0.05). TER was not significantly changed in response to PRV infection in *Kit^W-sh^/Kit^W-sh^* mice (Sh:–, n = 9, P>0.05), *Kit^W-sh^/Kit^W-sh^* mice reconstituted with *Kit^W-sh^/Kit^W-sh^* bone marrow (Sh:Sh, n = 7, P>0.05), TNF^−/−^ mice (n = 9, P>0.05), or TNFR1/2^−/−^ mice(n = 8, P>0.05). B) Evan's blue dye was administered i.v. to assess edema by quantifying dye extravasation into bladder tissues of sham-infected (white bar) and PRV-infected mice (black bars). Dye extravasation in B6 mice not receiving drug therapy (–) was significantly induced by PRV infection relative to sham infection (*P<0.05), and extravasation was significantly inhibited in TNF−/− mice (**P<0.05) or in mice treated with diphenhydramine (DHP, **P<0.05) but not inhibited in mice treated with ranitidine (RTD, P>0.05). C) Bladder TER was not influenced by antihistamine therapy. TER was quantified ex vivo for bladder of untreated mice (–) and mice receiving oral gavage therapy with diphenhydramine (H1) or ranitidine (H2). All mice infected with PRV (black bars) exhibited TER significantly reduced TER (*P<0.05) relative to sham-infected mice (white bar). Error bars depict SEM, and significance was determined by ANOVA.

We postulated that histamine contributes to bladder pathophysiology and thus induces pain only indirectly via an inflammatory cascade associated with tissue damage. To address this possibility, we examined bladder pathophysiology at the level of urothelial integrity by quantifying bladder TER ex vivo in an Üssing chamber (Fig. 6A), a physiologic parameter that correlates with mast cell-dependent bladder pathology in PRV cystitis [17]. PRV infection of wild type mice resulted in significantly compromised TER relative to untreated mice (P<0.05), whereas TER was unchanged in bladders of PRV-infected *Kit^W-sh^/Kit^W-sh^* mice. Stable TER following PRV infection was associated only with mice lacking mast cells or functional TNF signaling, confirming roles for mast cells and TNF in PRV-induced bladder pathophysiology. However, significantly compromised TER was observed in H1R^−/−^ and H2R^−/−^ mice, *Kit^W-sh^/Kit^W-sh^* mice reconstitiuted with HDC^−/−^ bone marrow (Fig. 6A, P<0.05), conditions that significantly reduced pain. These data argue against a role for histamine in bladder pathophysiology and instead suggest that mast cell histamine mediates pelvic pain directly.

### Histamine-associated pain is not coupled with inflammation

To exclude the possibility that histamine mediates pain and inflammation independent of pathophysiology, we assessed PRV-induced bladder inflammation using Evan's blue dye extravasation as a measure of edema [11], [13] (Fig. 6B). PRV infection of wild type mice resulted in significantly enhanced dye extravazation, whereas dye extravazation was largely abrogated in TNF^−/−^ mice (83.5% decrease, Fig. 6B). Since TNF^−/−^ mice exhibited normal pain responses (Fig. 2), these data suggest that pelvic pain is distinct from bladder inflammation and pathophysiology. In support of this possibility, the H2R antagonist ranitidine partially reduced edema (45.5%), had no protective effect on TER, but disproportionately relieved pain 92.9% (Figs. 6B, 6C, and 5, respectively). Finally, the H1R antagonist diphenhydramine reduced edema 95.0%, whereas PRV-induced loss of TER was unchanged, and pain was reduced 50.1%. Thus, pain is dissociated from the inflammatory effects of histamine.

## Discussion

Mast cells and histamine are known mediators of inflammatory responses, and it is assumed that inflammation is central to the local pain response. In PRV-induced cystitis, both genetic and pharmacologic data presented here suggest that mast cells mediate cystitis pain via H1R and H2R, whereas genetic data exclude TNF as a significant mediator of acute mast cell-dependent pelvic pain. These findings are further supported by the observations that pain precedes TNF-dependent pathophysiology by at least one day in murine PRV cystitis and that efficacy of antihistamines for pain is dissociated from effects on edema. The absence of pain responses in *Kit^W-sh^/Kit^W-sh^* mice indicates that mast cells are required for histamine-mediated pelvic pain, but *Kit^W-sh^/Kit^W-sh^* mice reconstituted with HDC^−/−^bone marrow exhibited diminished, albeit significant, pain. This raises the possibility that histamine from non-mast cell sources also contributes to pain, probably as a consequence of TNF-mediated inflammation, consistent with similar observations in airway models [32], [33]. Nonetheless, this study suggests that mast cells contribute to both pain and bladder inflammation and pathophysiology through the divergent actions of histamine and TNF, respectively. Finally, this study indicates that histamine receptors represent therapeutic targets for direct intervention in pain.

We observed isolectin B4-positive processes within the lamina propria, where mast cells accumulate in IC and PRV cystitis [4], [5], [11], [12], [17], suggesting that interactions between mast cell histamine and C fiber nociceptors mediate pelvic pain in this IC model. This possibility of C fiber involvement in IC pain is bolstered by the recent report that pain correlated with sensory nerve fiber density in patients with painful bladder syndrome (PBS, a clinical diagnosis for patients presenting with IC-like symptoms in the absence of cystoscopic evaluation) [34], [35]. Histamine may act on such bladder sensory nerves in IC/PBS because histamine metabolites are increased in IC urine, and mast cell histamine stimulates C fiber activity via H1R in an esophagitis model [10], [36]. Irritable bowel syndrome (IBS) is another chronic condition that resembles IC in that abdominal/pelvic pain occurs in the absence of overt pathology. In IBS, pain is correlated with activated colonic mucosal mast cells in proximity to nerves, and IBS colonic tissue extracts that contained elevated histamine excited rat nociceptors [37], [38]. These findings support the idea that mast cell histamine may mediate pain in multiple pelvic pain syndromes.

While this study does not preclude a role for chronic TNF-mediated pathophysiology in IC symptoms, our evidence for histamine-mediated pelvic pain provides a mechanistic understanding of previous clinical findings. The H2R antagonist cimetidine produced significant improvement in pain and nocturia in a limited trial of PBS patients [31]. Similar to our findings that PRV-induced pain mediated histamine is independent of TNF, symptom relief from cimetidine therapy was not associated with improved bladder pathology [39]. Pilot clinical studies yielded modest pain relief in IC patients receiving the old-line H1R antagonist hydroxyzine [40], but studies have not yet been conducted with newer generation H1R antagonists. In IBS, while clinical studies are lacking, anecdotal evidence (i.e., Internet searches) indicates that antihistamines are widely used by IBS patients. The colon modulates PRV-induced pelvic pain, pelvic organs generally exhibit neural-mediated crosstalk, and IBS is a co-morbidity of IC [13], [41] [42], [43], [44]. Therefore, these epidemiologic and animal model findings together suggest that H1R and H2R antagonists may have general application for the treatment of pelvic pain.

In summary, we find that the pelvic pain in an IC model is mediated by mast cell histamine acting on H1R and H2R. These findings support a general model for IC pelvic pain due to mast cell-sensory nerve interactions, and similar interactions may drive pelvic pain in IBS. Thus, antagonists of H1R and H2R are candidates for expanded clinical trials in the treatment of chronic pain conditions.

## Methods

### Animals

Adult female C57BL/6J mice, mice with targeted deletion of TNF (TNFKO, B6;129S6-Tnf^tm1Gkl^/J) or TNFR1 & TNFR2 (TNFRKO, B6;129S-Tnfrsfla^tm1Imx^Tnfrsflb^tm1Imx^/J) mice (10–14 weeks old) were purchased from Jackson Laboratory (Bar Harbor, ME). Mast cell-deficient *Kit^W-sh^/Kit^W-sh^* mice (B6.Cg-*Kit^W-sh^*/HNihrJaeBsmJ), mice targeted deletion of histamine receptor 1 (H1RKO, backcrossed 10 times onto C57BL/6) or histamine receptor 2 (H2RKO, backcrossed 8 times onto C57BL/6) mice (10–14 weeks old) were maintained in our breeding colony at Northwestern University. All experiments were performed using protocols approved by Northwestern University Animal Care and Use Committee. Mice were housed in containment facilities of the Center for Comparative Medicine and maintained on a regular 12:12 hour light:dark cycle with food and water *ad libidum*.

### Induction of neurogenic cystitis

Pseudorabies virus (PRV) was prepared and titrated as previously reported in Chen et al. Neurogenic cystitis was induced by injection of 2.29×10^6^ pfu Bartha's PRV in the abductor caudalis dorsalis (ACD) muscle with a 26 gauge Hamilton syringe while maintaining the animals under Isoflurane anesthesia. Ultraviolet-irradiated/heat inactivated PRV stocks were employed as negative control inocula in sham-treated mice. Both sham and PRV-infected mice were hydrated daily by subcutaneous injection of 3 ml saline in the shoulder region because previous experiments indicated that PRV altered fluid intake (Chen and Klumpp, unpublished observations).

### Behavioral Testing

Mice were tested prior to PRV administration (baseline), 1, 2, 3 and 4 post infection days (PID) after PRV inoculation. Referred hyperalgesia and tactile allodynia was tested using von Frey filaments applied to the abdomen [20], [45] and the plantar region of the hind paw. Mice were tested in individual Plexiglas chambers (6 cm×10 cm×12 cm) with a stainless steal wire grid floor (mouse acclimation period of ∼10 min prior to testing). Frequency of withdrawal responses to the application of von Frey filaments to the abdomen was tested using five individual fibers with forces of 0.04, 0.16, 0.4, 1 and 4 grams (Stoelting, USA). Each filament was applied for ∼1 second with an inter-stimulus interval of 2–5 s for a total 10 times, and the hairs were tested in ascending order of force. Stimulation was confined to the lower abdominal area in the general vicinity of the bladder and care was taken to stimulate different areas within this region to avoid desensitization or “wind up” effects. Three types of behaviors were considered as positive responses to filament stimulation: (1) sharp retraction of the abdomen; (2) immediate licking or scratching of the area of filament stimulation; or (3) jumping.

Tactile allodynia was tested on the plantar region of the hind paw using von Frey filaments with forces of 0.04, 0.16, 0.4, 1 and 4 grams. The median 50% withdrawal threshold was assessed using the up-down method where testing was started with 0.04 g filament applied perpendicularly to the plantar surface of the hind paw until the filament bent slightly. Filaments were tested in ascending order until a positive response was observed. A positive response to the filament was defined as either a sharp withdrawal of the paw or licking of the test paw. When a positive response was recorded the next weaker filament was applied, and if a negative response was observed, then the next stronger filament was applied.

Spontaneous behavior was recorded (Sony VAIO USB camera) for five minutes in a clear plastic open field chamber (18×29×12 cm) at 96 hours after PRV infection and scored for rearing, grooming and cage crossing to assess general activity.

### Capsaicin Treatment

Capsaicin (50 µl) was administered as 0.3% solution (dissolved in 10% ethanol, 10% Tween 80 and 80% saline) instilled into the colon via a Hamilton syringe catheter (rounded-tip needle 3.8 cm long) while the mouse was maintained under Isoflurane anesthesia. All mice were tested for referred hyperalgesia and tactile allodynia using von Frey filaments before and 20 min after capsaicin treatment.

### Antihistamine Treatment

Antihistamine treatment (10 mg/kg) was initiated 1 hour prior to PRV inoculation and was continued every 24 hours until PID 4. Diphenhydramine, ranitidine, thioperamide (Sigma, St. Louis, MO) or a combination of diphenhydramine/ranitidine was administered by oral gavage (Hamiltion syringe with a rounded tip needle 2.5 cm long). PRV-infected mice and sham controls were gavaged with saline until PID4. All mice were tested for referred hyperalgesia and tactile allodynia using von Frey filaments before PRV (baseline) and PID1-4.

### Mast cell reconstitution

Bone marrow was collected from the femurs of either wild-type female C57BL/6J or *Kit^W-sh^/Kit^W-sh^* mice. Cell suspensions were generated by trituration, red blood cells were lysed, and the remaining whole bone marrow cells were harvested by centrifugation at 1200 rpm for 5 min. The resulting bone marrow cells were then resuspended in 1x phosphate buffered saline for injection. Female 4-6 week old sash mice were injected retro-orbitally with 200 µl PBS containing 1×10^7^ whole bone marrow cells from C57BL/6J mice, *Kit^W-sh^/Kit^W-sh^* mice, or saline alone. All mice were tested 8 weeks after reconstitution for referred hyperalgesia and tactile allodynia using von Frey filaments before PRV (baseline) and PID1-4. Bladders were excised, and mast cells were quantified in paraffin sections following toluidine blue staining.

### Acidified toluidine blue staining

Tissue sections were deparaffinized, rehydrated, and stained with acidified toluidine blue to visualize mast cells, as described. Briefly, tissue sections were immersed in 0.5% potassium permanganate. After rinsing in distilled water, sections were immersed in 2% potassium metabisulfite, incubated in tap water, and washed with distilled water. Lastly, sections were immersed in 0.02% toluidine blue in 0.25% glacial acetic acid, pH 3.2. To quantify mast cells, toluidine blue-stained cells were counted in two non-adjacent tissue sections and reported as the mean of sections for each animal.

### Isolectin B4 staining and image analysis

C57BL/6 mouse bladders were fixed in 4% paraformaldehyde for 10 minutes and cryoprotected in 10% sucrose for 1 hour and 30% sucrose overnight. Bladders were mounted in OCT, cut into 20 µm sections, and dried at 37°C for 1.5 hours. Sections were incubated with 10 µg/ml fluorescein-conjugated *Griffonia(Bandeiraea) Simplicifolia* Lectin I, isolectin B4 (Vector Laboratories), for 1 hour at room temperature. As a negative control, additional sections were incubated with isolectin B4 that was pre-incubated with 500 mM D-galactose for 1 hour prior to staining. Sections were washed and counterstained with 4′,6-diamidino-2-phenylindole (DAPI). Images were collected using a Leica DMIRE2 inverted microscope using Volocity 4.0 software (Improvision) for acquisition and analysis. A Z-stack of 30 µm total thickness was taken at 0.1 µm intervals and was deconvolved using the Volocity Deconvolution module (25 iterations). The C fiber was identified by pixel intensity and its skeletal length was measured using the Volocity Measurements module.

### RT-PCR

Whole bladders and sections of the colon were removed from five wildtype female C57BL/6 mice. Total RNA was extracted from the samples using Trizol (Invitrogen) extraction according to manufacturer's directions. RNA was reverse transcribed using an iScript cDNA Synthesis Kit (Biorad) according to manufacturer's directions. Quantitative Real Time PCR analysis was performed using iQ SYBR Green Supermix (Bio-Rad) in a MJ Research Chromo 4 thermocycler using the following primer sequences. *Hrh1* (Forward) 5′-TCG TCA TGC CCA TGA ACA TCC TCT-3′ and (Reverse) 5′-TGA AGC ACG GGT CTT GGT TCG ATA-3′; *Hrh2* (Forward) 5′-CGC GTT GCC ATC TCT TTG GTC TTT-3′ and (Reverse) 5′-TCG TTG ACC TGC ACT TTG CAC TTG-3′; *Hrh3* (Forward) 5′-TGG TGT CCT CCC TAA TGC AAA CCT-3′ and (Reverse) 5′-ATT AAG GAA GAG ACA GCG GCA GCA-3′; *Hrh4* (Forward) 5′-AGG ACT GTG AGC CTG GCT TTG TTA-3′ and (Reverse) 5′-AAG CCA CAG AGA TGA CAG GAA GCA-3′; *L19* (Forward) 5′-CCA TGA GTA TGC TCA GGC TTC AGA-3′ and (Reverse) 5′-TAC AGG CTG TGA TAC ATG TGG CGA-3′. Histamine receptor mRNA levels were normalized to ribosomal protein L19 mRNA levels.

### Bladder TER

Bladders were harvested, rinsed with PBS, and dissected exposing the luminal surface. The tissue was carefully secured in an Üssing chamber (model CHM8; World Precision Instruments Inc., Sarasota, FL) and filled with 0.1 M KCl. Using electrodes filled with 0.1 M KCl solution, TER measurements were obtained with a EVOMX epithelial voltohmeter (WPI Inc.). Bladder TER was determined based upon resistance after stabilazation (∼5 min) and reported as the product of the stabilized TER and the tissue area (0.126 cm^2^) and reported as Ωcm^2^. All measurements were performed at room temperature.

### Evans blue extravasation

Vascular permeability was assessed by measuring Evans blue dye extravasation. Evans blue (30 mg/kg in phosphate buffered saline) was administered intravenously via the tail vein and allowed to circulate for 30 minutes prior to sacrifice. Individual bladders or colons were harvested, weighed, placed into 1 ml formamide (Sigma) and incubated for 24 hours at 60°C. Extravasation was quantified by measuring absorbance (A_620_) relative to a standard curve of dye.

### Statistical analyses

Results were expressed as mean±SEM and analyzed for statistical significance by a one-way ANOVA followed by a post-hoc test comparison using Dunnet's multiple comparison. A value of p<0.05 was considered statistically significant.

## Supporting Information

Figure S1 Bladder and colon express histamine receptors. Histamine receptor mRNAs were quantified by real-time RT-PCR of total RNA prepared from bladder (A) or colon (B) of wild type B6 mice (n  =  5). Primers were specific for mouse ribosomal protein L19 or receptors H1R, H2R, H3R, or H4R.
(0.36 MB EPS)

Table S1 (0.05 MB DOC)

Table S2 (0.05 MB DOC)

Table S3 (0.05 MB DOC)

Movie S1 Deconvolution microscopy and 3D reconstruction of a bladder nociceptor labeled by staining with isolectin B4.
(0.67 MB MOV)
