# Supplementary material for: Mast Cell-Derived Histamine Mediates Cystitis Pain
Source: PLoS One. 2008 May 7;3(5):e2096. doi: 10.1371/journal.pone.0002096 (PMC2346452; doi:10.1371/journal.pone.0002096)
Supplement: Table S1 — (0.05 MB DOC) [file pone.0002096.s002.doc]

Table S1. Paw sensitivity determined by 50% Threshold (g, *p<0.05).
Group	Baseline	PID 1	PID 2	PID 3	PID 4	
Sham	1.03±0.23	0.94±0.25	0.91±0.26	1.15±0.29	1.16±0.33	
PRV	1.05±0.26	1.15±0.31	1.24±0.28	1.27±0.23	1.29±0.23	
KitW-sh/KitW-sh	1.26±0.26	1.30±0.27	1.31±0.28	1.38±0.36	1.36±0.27	
KitW-sh/KitW-sh :WT/WT	1.57±0.28	1.40±0.17	1.42±0.24	1.58±0.23	1.46±0.24	
KitW-sh/KitW-sh :HDC/HDC	1.61±0.28	1.64±0.24	1.45±0.24	1.85±0.35	1.50±0.33	
KitW-sh/KitW-sh :KitW-sh/KitW-sh	1.60±0.34	1.39±0.24	1.61±0.33	1.53±0.28	1.54±0.34	
KitW-sh/KitW-sh :PBS/PBS	1.28±0.26	1.59±0.38	1.69±0.38	1.46±0.41	1.31±0.38	
TNF-/-	1.34±0.35	1.10±0.36	1.28±0.41	1.18±0.35	1.02±0.28	
TNFR1/2-/-	1.47±0.32	1.45±0.32	1.37±0.23	1.43±0.22	1.47±0.31	
H1R-/-	1.65±0.44	1.56±0.46	1.46±0.26	1.59±0.45	1.51±0.48	
H2R-/-	1.73±0.65	1.63±0.60	1.47±0.37	1.57±0.30	1.73±0.56	
Diphenhydramine (H1)	2.18±0.39	1.94±0.27	2.03±0.35	1.89±0.31	2.18±0.38	
Ranitidine (H2)	1.60±0.38	2.08±0.39	1.73±0.32	1.88±0.38	1.79±0.30	
Thioperamine (H3/4)	2.08±0.47	1.80±0.44	1.60±0.31	1.80±0.44	1.93±0.55	
H1 & H2	2.02±0.32	2.09±0.29	1.91±0.27	2.22±0.24	1.91±0.27	
Saline (PRV)	2.12±0.33	1.73±0.31	1.99±0.39	1.96±0.30	1.73±0.30	
