# Supplementary material for: Mast Cell-Derived Histamine Mediates Cystitis Pain
Source: PLoS One. 2008 May 7;3(5):e2096. doi: 10.1371/journal.pone.0002096 (PMC2346452; doi:10.1371/journal.pone.0002096)
Supplement: Table S2 — (0.05 MB DOC) [file pone.0002096.s003.doc]

Table S2. Spontaneous Behaviors (*p<0.05).
Group	Crossing	Rears	Grooming	
Sham	61.0±7.7	37.1±2.4	3.2±0.3	
PRV	54.1±6.4	36.8±2.9	3.2±0.5	
KitW-sh/KitW-sh	39.7±8.5	21.9±4.5*	3.8±0.6	
KitW-sh/KitW-sh :WT/WT	37.3±4.5	20.0±3.3*	4.5±0.6	
KitW-sh/KitW-sh :KitW-sh/KitW-sh	38.0±4.2	22.7±4.6*	3.3±0.7	
KitW-sh/KitW-sh :PBS/PBS	37.5±3.2	14.4±2.4*	3.8±0.5	
TNF-/-	39.0±5.9	20.7±3.6*	3.8±0.5	
TNFR1/2-/-	59.1±4.5	44.1±4.3	1.8±0.6*	
H1R-/-	50.4±11.7	27.8±5.5	3.4±0.6	
H2R-/-	64.8±10.0	27.8±5.9	3.0±0.7	
Diphenhydramine (H1)	51.0±7.1	32.6±4.6	3.7±0.5	
Ranitidine (H2)	70.7±9.1	31.7±2.6	2.9±0.4	
Thioperamine (H3/4)	46.8±6.8	27.8±4.6	4.2±0.8	
H1 & H2	61.4±6.3	33.0±4.3	2.5±0.5	
Saline (PRV)	50.5±6.4	28.3±3.9	3.3±0.6	
