# Supplementary material for: Mast Cell-Derived Histamine Mediates Cystitis Pain
Source: PLoS One. 2008 May 7;3(5):e2096. doi: 10.1371/journal.pone.0002096 (PMC2346452; doi:10.1371/journal.pone.0002096)
Supplement: Table S3 — (0.05 MB DOC) [file pone.0002096.s004.doc]

Table S3. Body mass during infection (*p<0.05).
Group	Baseline	PID 1	PID 2	PID 3	PID 4	
Sham	18.3±0.6	17.3±0.9	17.4±0.8	17.6±0.7	17.9±0.6	
PRV	18.4±0.5	17.4±0.8	17.7±0.7	17.7±0.6	18.0±0.6	
KitW-sh/KitW-sh	23.5±0.7	22.9±0.8	22.9±0.8	22.9±0.8	22.6±0.8	
KitW-sh/KitW-sh :WT/WT	22.8±0.7	22.7±0.7	22.6±0.7	22.8±0.7	22.4±0.8	
KitW-sh/KitW-sh :HDC/HDC	19.8±0.6	19.8±0.6	19.6±0.5	19.5±0.6	19.3±0.7	
KitW-sh/KitW-sh :KitW-sh/KitW-sh	23.6±0.5	23.6±0.6	23.5±0.5	23.9±0.5	23.9±0.5	
KitW-sh/KitW-sh :PBS/PBS	23.1±0.6	23.2±0.6	23.1±0.6	23.2±0.6	23.1±0.6	
TNF-/-	19.8±0.3	19.8±0.4	19.7±0.4	19.8±0.4	19.5±0.4	
TNFR1/2-/-	21.7±0.5	21.7±0.4	22.4±0.4	23.1±0.3	23.2±0.3	
H1R-/-	21.3±0.3	21.2±0.3	21.3±0.4	21.6±0.4	21.6±0.4	
H2R-/-	18.7±0.4	18.8±0.5	18.8±0.4	18.9±0.4	18.9±0.3	
Diphenhydramine (H1)	20.3±0.4	19.3±0.4	19.6±0.4	19.9±0.4	19.7±0.4	
Ranitidine (H2)	20.1±0.4	19.6±0.4	19.9±0.4	19.8±0.4	19.4±0.5	
Thioperamine (H3/4)	19.2±0.2	19.2±0.4	18.4±0.4	19.3±0.4	18.5±0.3	
H1 & H2	19.2±0.5	19.3±0.5	18.9±0.6	20.3±0.4	19.7±0.3	
Saline (PRV)	20.1±0.5	20.0±0.5	20.2±0.5	20.3±0.4	20.1±0.4	
